# Body Temperature Responses During Phases of Work in Human Remains Detection Dogs Undergoing a Simulated Deployment

**DOI:** 10.3390/ani10040673

**Published:** 2020-04-13

**Authors:** Janice Baker, Mallory DeChant, Eileen Jenkins, George Moore, Kathleen Kelsey, Erin Perry

**Affiliations:** 1 Veterinary Tactical Group, VTG Canine Sports Medicine and Rehabilitation Clinic, Vass, NC 28394, USA; jbaker@vettacgroup.com; 2 Department of Animal Science Food and Nutrition, Southern Illinois University, Carbondale, IL 62901, USA; Mallory.Dechant@ttu.edu; 3 First Year Graduate Veterinary Education Program, Public Health Activity-Fort Bragg, Fort Bragg, NC 28310, USA; eileen.jenkinsdvm@gmail.com; 4 Department of Veterinary Administration, Purdue University, West Lafayette, IN 47907, USA; gemoore@purdue.edu; 5 Working Dog Enterprises, Sturgeon, MO 65284, USA; kkelsey@workingdogenterprises.com; 6 Department of Animal Science Food & Nutrition, Southern Illinois University, Carbondale, IL 62901, USA

**Keywords:** working dogs, travel, thermal stress

## Abstract

**Simple Summary:**

Working dogs are frequently transported via crates in vehicles to various deployment scenarios. Body temperature increase associated with exercise is common but has not been assessed throughout the entire work cycle for dogs during a deployment scenario. We measured continuous temperature changes for dogs throughout an entire day of search operations including each stage of the work cycle, namely waiting-to-work, active work, and post-work recovery. We found that dogs did not increase in temperature while waiting-to-work but temperature did increase during the period of active work. Additionally, when dogs were returned to vehicles for crate and rest during the post-work recovery period, body temperatures continued to increase. We suggest that post-work recovery in the vehicle should be further investigated to better manage dogs through continuing search operations.

**Abstract:**

Body temperature responses were recorded during phases of work (waiting-to-work in close proximity to search site, active work in a search site, and post-work recovery crated in vehicle) in human remains detection dogs during search training. State or federally certified human remains detection dogs (*n* = 8) completed eight iterations of searching across multiple novel search environments to detect numerous scent sources including partial and complete, buried, hidden, or fully visible human remains. Internal temperature (Tgi) of the body was measured continuously using an ingestible thermistor in the gastrointestinal tract. Mean total phase times were: waiting-to-work: 9.17 min (±2.27); active work: 8:58 min (±2:49); and post-work recovery: 24:04 min (±10.59). Tgi was impacted by phase of work (*p* < 0.001) with a small increase during active work, with mean peak temperature 39.4 °C (±0.34 °C) during that period. Tgi continued to increase for a mean of 6:37 (±6:04) min into the post-work recovery phase in the handler’s vehicle with a mean peak Tgi of 39.66 °C (±0.41 °C). No significant increase in temperature was measured during the waiting-to-work phase, suggesting behaviors typical of anticipation of work did not appear to contribute to overall body temperature increase during the waiting-to-work recovery cycle. Continued increase of gastrointestinal body temperature several minutes after cessation of exercise indicates that risk of heat injury does not immediately stop when the dog stops exercising, although none of the dogs in this study reached clinically concerning body temperatures or displayed any behavioral signs suggestive of pending heat injury. More work is needed to better understand the impact of vehicle crating on post-work recovery temperatures in dogs.

## 1. Introduction

Working dogs and other canine athletes often follow a cycle of waiting or staging to work, active work, and post-work recovery. During actual search deployments, dogs will often stage while waiting-to-work in a vehicle or crate near the search site. Similarly, they are often returned to this or a similar environment immediately following active work. Little is known about the response of body temperature throughout these phases continually. Body temperature is a particularly important physiologic parameter to monitor during these cycles, combined with observation of potential changes in behavior, to both mitigate life-threatening heat injury as well as safely maximize the duration of work the canine can perform. Multiple studies have characterized body temperature changes of dogs during exercise and during short recovery periods following exercise [1,2,3,4,5]. However, body temperature alone, collected at a single time point, fails to accurately predict or define heat injury. Numerous studies have shown conditioned dogs reaching temperatures of over 41 °C, and as high as over 42 °C during exercise, with no adverse effects [3,6,7,8]. Thus, other ways of looking at body temperature other than a single peak temperature as a measure of thermal status in dogs should be investigated. In addition, studies on dogs in controlled laboratory settings may not accurately represent physiological responses of trained working dogs under field conditions which typically include transportation and crating within a vehicle.

Many studies have investigated changes in body temperature of various types of working and sporting dogs during active exercise [1,2,3,4,5,6,9,10,11,12]. Diviero et al., in a study on avalanche search and rescue dogs at an approximately ambient temperature of −10 °C, showed an increase in 0.58 °C that could be attributed to environment and anticipation stress, measured after helicopter transport and lowering from the helicopter by harness with the handler. This study showed that after significant increases in rectal temperature in anticipation of work, rectal temperatures remained relatively constant with a mean of 39.15 °C throughout their approximately 10-min search. Rovira et al. studied changes in search and rescue (SAR) dogs in more moderate (approximately 21 °C) temperatures, demonstrating that search and rescue dogs peaked at a mean rectal temperature of 40.64 °C and did not show a significant drop in temperature throughout the entire 30-min recovery period. However, few data are available regarding the impact of deployment conditions on the temperature of working dogs throughout a day of work or training in the field from waiting or staging to work, active work, and in post-work recovery while crated in a vehicle.

In this study, our objective was to evaluate the hypothesis that body temperature responses in dogs would follow distinct, and thus predictable, patterns throughout the different phases of work, using a waiting-to-work, active work, and post-work recovery cycle, with measurements obtained in the typical environment for that phase, whether in a crate, the active search site, or recovering in the handler’s vehicle. Furthermore, we hypothesized that dogs would have a rise in temperature associated with physical behaviors in anticipation of work with potential continued increase throughout traditional work cycles and the post-work recovery phase. Knowledge of these patterns could be beneficial in understanding expected body temperature changes in dogs during work, help mitigate adverse effects such as heat injury, and better understand patterns of thermoregulation in the canine athlete.

## 2. Materials and Methods

### 2.1. Animals and Diet

Ten urban search and rescue (USAR) dogs that were trained and/or certified to a national testing standard in human remains detection were initially recruited for the study. Participants were standardized to a commercially available diet (Canidae Pure) for 30 days prior to the study. Canines had ad libitum access to water throughout the experimental period and were examined by a licensed veterinarian immediately prior to inclusion of the study. Exclusion criteria included: medications other than scheduled flea, tick, heartworm, or internal parasite preventive, intolerance veterinary exam, or in close proximity to persons other than the handler (aggression, obvious anxiousness, significant effort to avoid the examiner or get away from handler’s control), demonstrated aggression towards other dogs, or physical exam findings of lameness or signs of potentially undiagnosed illness. Upon the veterinary exam, one canine displayed excessive aggression and was removed from the study. Another canine was also removed from the analysis due to a suspected undiagnosed metabolic disorder. Thus, the results and discussion presented are for a total of eight dogs. Care and handling of animals used in this study was approved by Southern Illinois University Animal Care and Use Committee, Animal Use Protocol 16-037. Physical characteristics for participating dogs are presented in Table 1.

### 2.2. Study Design

**Figure 1:**
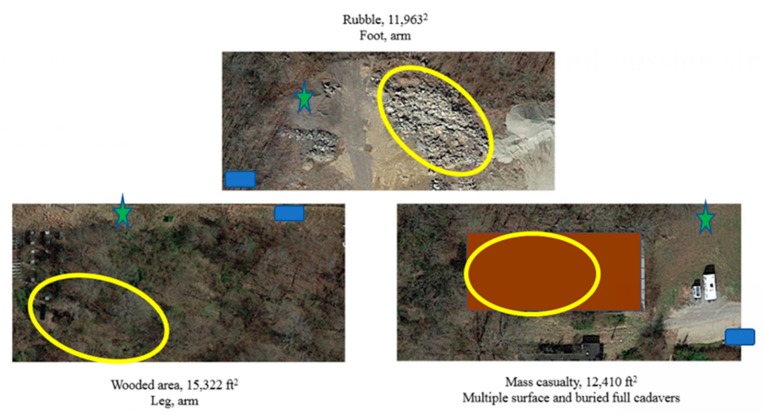
Search environments for human remains detection dogs during a simulated day of tactical deployment operations. Deployment operations consisted of four rotations across three environments with two scenarios at each search environment for a total of eight complete work cycles (WW = waiting-to-work, AW = active work, PWR = post-work recovery) for each canine. Green star = waiting-to-work; yellow circle = active work; blue rectangle = post-work recovery; brown rectangle = For purposes of donor privacy, the mass casualty area has been blurred so as to avoid unintentional identification of donors.

Canines were randomly assigned to rotate through six deployment search sites in which whole, or partial human remains in various states of decomposition at a forensic anthropology field laboratory were situated. Searches 1–6 were all unique and locations were unknown to all study participants. During the final rotation of the day, dogs repeated the search from their first assigned site (searches 7–8) for a total of eight search iterations for each dog. All dogs arrived at their assigned search site at the same time in the morning. All canines started their work cycle sequence in the waiting-to-work (WW) phase (1 dog at each site), and all dogs rotated sequentially through the phases and sites throughout the day over approximately 9 h, with a mid-day break for all study participants of 1 h. Search site areas ranged from 5100–18,800 ft^2^ with terrain typical of disaster deployments including a grass field, fallen building rubble, a mass casualty scenario, and wide area with trees, and mild to moderate hills. Phases of work for each site were defined as: WW = waiting-to-work in staging area with exposure to olfactory, auditory, and limited visual stimuli and the dog crated next to handler approximately 10–15 m from the search site; AW = active work off leash within the search site; PWR = post-work recovery crated in the handler’s vehicle with no exposure to visual or olfactory stimuli from the search sites approximately 75–100 m from the search site (Figure 1).

**Figure 2:**
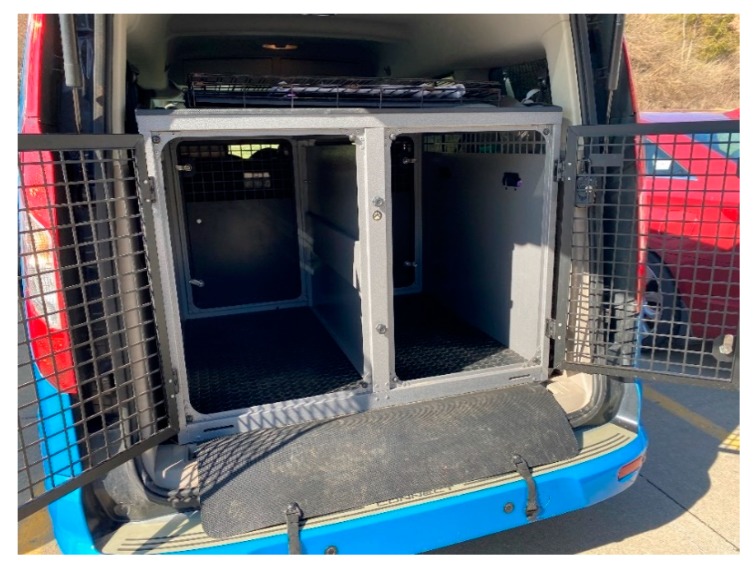
Cargo van with dual crate system and removable center divider.

**Figure 3:**
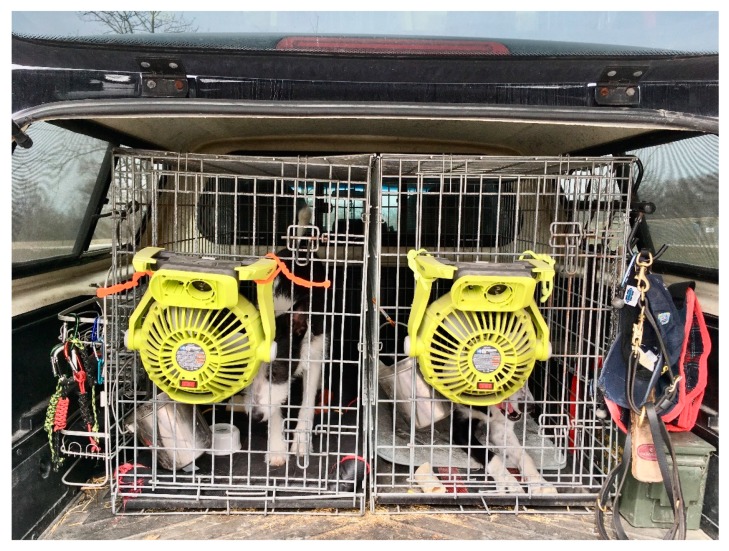
Pick-up truck with camper shell and dual crate system with battery operated mounted fans.

Handler vehicles included pick-up trucks with camper shells (2), pick-up trucks with open bed (2), SUVs (3), and a minivan (1). No dogs were exposed to air conditioning and doors and/or windows were opened to maximize air flow in all vehicles except the open-backed pick-up trucks, in which dogs were crated. Due to the small number of vehicles, and variety of vehicles, differences of dogs’ body temperature between vehicle types were not evaluated. Dog-handler teams worked for 7.5 h across all sites. Handler vehicles were typical of those used in the working dog industry as evidenced by the examples provided below (Figure 2 and Figure 3).

### 2.3. Animal Performance and Sample Collections

Environmental temperature and humidity were recorded hourly (National Oceanic and Atmospheric Administration (NOAA), Carbondale/Murphysboro Southern Illinois Airport, 5.8 miles from study location) [13] for the study date in April (Table 2).

**Figure 4:**
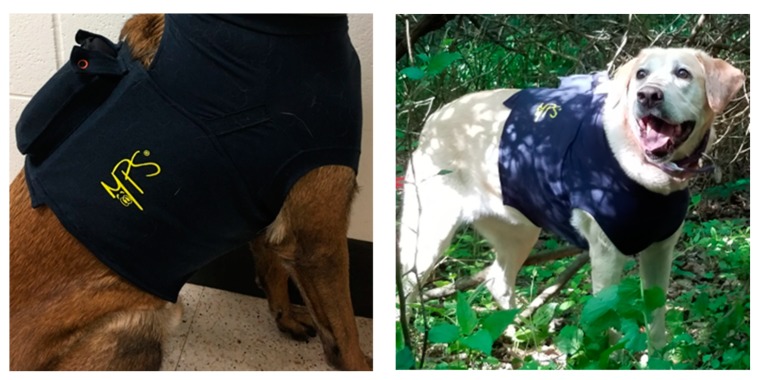
Medical vest worn by human remains detection canines during simulated tactical deployment operations.

Gastrointestinal body temperature (Tgi) was measured in the gastrointestinal tract utilizing CorTemp ^®^ Ingestible Sensor: 262kHz (HT150002; HQ, Inc.; Palmetto, FL, USA) which recorded the gastrointestinal temperature in 10 s increments. CorTemp^®^ Ingestible Sensors (HQ, Palmetto, FL, USA) have been previously utilized for core body temperature studies in canines [1,6,11,14,15]. Sensors were ingested 45 min (±15) prior to initiation of the study. CorTemp^®^ Data recorders were affixed within a lightweight, non-restricting medical vest (Medical Pet Shirt International BV, MPS Protective Top Shirt 4 in 1, Zoetermeer, Netherlands) worn by each dog for the duration of the study (see Figure 4) All canines were acclimated to the vests for 14 days prior to the start of the study by wearing them during training or search activities for 60 min each day.

Iteration baseline temperature was considered the first temperature reading of each WW period (eight measures per study participant). Peak temperature was noted as the highest recorded temperature at any point in each complete WW, AW, and PWR cycle (eight measures per study participant). Dogs were allowed to drink water in the post-work recovery phase which is known to momentarily drop the temperature recorded by the ingestible thermistor. Thus, body temperature recordings (*n* = 4) under 35 °C (95 °F) were excluded from analysis.

### 2.4. Statistical Analysis

Numerical data were assessed for a normal distribution by the Shapiro–Wilk test. Normally distributed numerical data are summarized as mean (±SD). Non-independent parametric numerical data in the same dog and comparisons of highest Tgi in a phase with the initial Tgi for that phase were compared with repeated measures ANOVA followed by paired t-tests. Peak temperature was also compared by ANOVA, accounting for repeated measures in dogs, across iterations to assess impact of time of day on internal temperature. Correlation between time spent in an AW phase and the time to reach peak temperature in the accompanying PWR phase was assessed by Spearman’s rank correlation coefficient (rho). Proportional frequency of the occurrence of iteration peak Tgi in the three phases was compared by the χ^2^ test of association. Statistical analysis was performed using commercially available software (STATA SE, v. 15.1, StataCorp, College Station, TX, USA) with statistical significance established at *p* < 0.05.

## 3. Results

Our dataset yielded 190 complete sets from 192 possible with each set representing one of three phases (WW, AW, or PWR) from one of eight search rotations for each of the eight dogs. Incomplete datasets resulted from failure of the CorTemp system to capture or report >3 min of temperature in one dog, impacting two datasets which were excluded from analysis. Thus, the data presented are for 5027 of the 5088 data points collected. Mean ambient temperature during the study period was 19.9 °C (±4.23). Relative humidity was 83.2% (±0.162) with a mean ambient temperature of 23.3 °C for the morning iterations (9:00 am–12:00 pm) and afternoon temperatures (approximately 1:00 pm–4:45 pm) of 15.6 °C. The sky was mostly clear in the morning until approximately 12:00 pm with complete cloud cover the remainder of the day. Mean total phase times were: WW: 9.17 (±2.27) min; AW: 8:58 (±2.49) min; and PWR: 24:04 (±10.59) min. The fastest dog finished the search operations with a mean of 6.43 min spent in AW (±3.09) and a peak temperature of 38.9 °C. Conversely, the slowest dog finished the search operations with a mean of 10.50 min spent in AW (±4.07) and a peak temperature of 40.0 °C.

Mean Tgi measured during staging at the “check-in” area was 38.4 °C. Upon arrival at each search site, mean Tgi for the iteration baseline at the beginning of the work cycle was 39.07 °C. The highest measured Tgi of any dog during the study was 40.6 °C occurring in the post-work recovery phase of the second iteration of the day. Mean peak Tgi of all dogs was 39.66 (± 0.40) °C across all iterations and phases of work. Mean increase (Tgi) was 0.66 °C (±0.19) from mean iteration baseline of 39.1 °C (±0.377). Peak Tgi was significantly more likely to occur in the PWR phase than the AW phase (χ^2^ = 166.3; *p* < 0.001).

**Figure 5:**
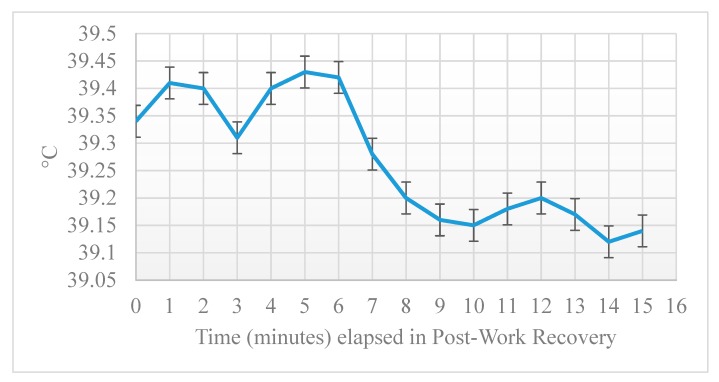
Mean gastrointestinal temperature (Tgi) (±SE) for human remains detection dogs during post-work recovery in handler vehicles.

Tgi was impacted by phase of work with a small but steady increase during active work (27 °C) with a high temp of 39.4 °C (±0.35) during AW and continuing to increase by 0.66 ºC over a span of 7:37 (±6:04) min following cessation of exercise during the post-work recovery phase (Figure 5). Mean Tgi across all phases is presented in Table 3.

**Figure 6:**
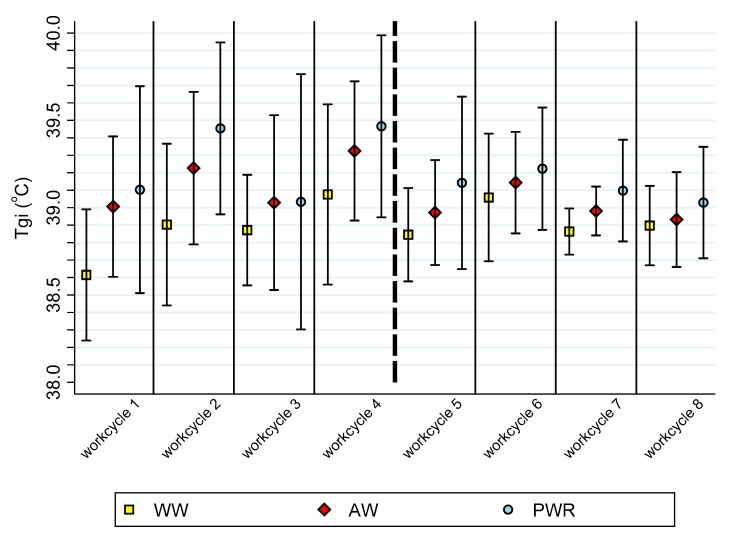
Mean (±SD) internal temperatures (Tgi °C) across eight search iterations representing three phases of work (waiting-to-work, active work, post-work recovery) in a single day of simulated deployment operations.

Time spent working was inversely related to the time required to reach peak temperature in the recovery phase (Spearman’s rho = −0.517; *p* < 0.0001). In 54.2% of the iterations, the peak temperature was followed by a drop in measured temperature occurring between 6:37 and 10 min following cessation of exercise. The mean peak Tgi during the waiting-to-work (WW) phase did not significantly differ from the WW phase baseline Tgi (*t* = −1.472; *p* = 0.073). Time of day, which was associated with greatly varying environmental temperatures and a large drop in temperature as the day progressed, did not impact time or phase when the peak temperature occurred, (*F* = 1.47; *p* = 0.236). Mean Tgi across all work cycles throughout all searches is displayed in Figure 6.

## 4. Discussion

### 4.1. Waiting-to-Work

Unlike previous studies on the effect of anticipation of work [4,10], body temperatures in the dogs did not increase during the waiting-to-work phase despite the fact that dogs were exposed to auditory, olfactory, and some visual cues of the working environment. Gillette et al. showed increases in rectal temperature, heart and respiratory rates of Greyhounds in anticipation of a trained exercise event. Dogs that were positioned to watch other dogs continued to show increases in rectal temperature and heart rate, even though they were not participating in the exercise. In a study on avalanche search dogs, Diviero et al. showed an increase in 0.58 °C that could be attributed to environment and anticipation stress, measured after helicopter transport and lowering from the helicopter by harness with the handler. This study showed that after significant increases in rectal temperature in anticipation of work, rectal temperatures remained relatively constant with a mean of 39.15 °C throughout their approximately 10-min search. This may have been affected by the fact that the dogs were working in snow at ambient temperature ranges between −8.5 °C and −10.4 °C with wind chill temperature of −29 °C. Dogs in the more moderate temperature study were noted to reach a mean peak of 40.64 °C after 20 min of exercise [5].

It is not clear why we did not see similar anticipatory responses in this group of dogs. Differences in training, acclimation to their environment, or other more complex behavioral aspects may have played a role in this difference, and further study on the effect of work anticipation on physiologic parameters is warranted. Dogs that are well acclimated to deployment conditions are likely conditioned to waiting and it is possible that this may have been a mitigating factor for the temperature increase reported in prior studies [4,10].

### 4.2. Active Work

The dogs in our study demonstrated a consistent rise in gastrointestinal temperature during active work, with a mean increase of 0.39 °C, which was small in comparison to other studies in exercising dogs. For example, Steiss et al. demonstrated a change in rectal temperature up to 3 °C in Labrador Retrievers doing less than five minutes of mild to moderate exercise, while Matwichuk et al. noted a mean increase of 2.4 °C in Labrador Retrievers after ten minutes of strenuous exercise [16]. The highest temperature of any individual dog during active work reached 40.24 °C. The continued rise in body temperature is consistent with previous studies that measured Tgi using the same instrument system [1,3,6,7,11]. However, the Baker and Davis study showed a trend toward temperature plateau during active exercise in dogs when they were in a highly conditioned state, but a continual rise throughout active work when unconditioned. Nazar et al. demonstrated that unconditioned dogs (those that had been restricted from activity for eight weeks) had a more rapid rise in rectal temperature in response to exercise than they did after they were in a conditioned state; although their endurance (time to exhaustion) increased by 119% after conditioning, they showed signs of exhaustion at similar rectal temperatures as when activity was restricted. The plateau effect reported in studies of exercising dogs [1,17] was not seen during the active work phase in our study, possibly due to the shorter working times in our study, and also possibly due to the stark differences between work requirements and selective breeding for specific physical traits between racing sled dogs and search and rescue dogs. In addition, while it was assessed that this group of dogs was fit for duty, we did not attempt to control or quantify the degree of physical fitness or conditioning regiments between individual dogs, so lack of temperature plateau during active work cannot be adequately assessed here.

### 4.3. Post-Work Recovery

Peak temperature following exercise was significantly more likely to occur in the PWR phase than the AW phase with 95.16% recorded peaks occurring in the PWR phase several minutes following cessation of exercise (mean 6.37 min). Mean peak Tgi was 39.66 °C. This is similar to results described by Pellegrino et al., O’Brien et al., and Rovira et al. Pelligrino et al. demonstrated that rectal temperatures peaked at 40.5 °C five minutes after cessation of sprint exercises in Greyhounds, while temperatures of military working dogs in the O’Brien et al. study continued to rise for 8 to 12 min following the end of exercise, demonstrating that continued metabolic heat production was greater than cooling by normal thermoregulatory methods (i.e., panting) and passive environmental cooling. Rovira et al. demonstrated that search and rescue dogs peaked at a mean rectal temperature of 40.64 °C and did not show a significant drop in temperature throughout the entire 30-min recovery period, suggesting that in those dogs, continued metabolic heat production was equal to thermoregulatory and passive environmental cooling combined throughout the recorded post-work recovery phase. Recovery phase housing details are not consistently provided in prior studies and differences in crating, vehicle transport, or return to kennels may contribute to contradictory results.

A small dip in mean temperature was noted at three minutes post-recovery. (Figure 4). This dip was very small, less than 0.1 C total, and not considered a clinically significant change in temperature. The cause of this dip is unknown and may appear exaggerated in significance based on the small unit increments used in the chart. Influence of water ingestion or position of the thermistor in the GI tract was ruled out by examining the mean and median temperatures at the beginning of the day, when the thermistor would be in the stomach, and at the end of the day, when the thermistor would be too far along the GI tract to be influenced by water ingestion. Median temperature at three minutes post-work recovery was actually higher in the morning than the afternoon, suggesting this dip was not the result of ingestion of water. Surprisingly, there was an inverse relationship between time of active work and time until peak temperature occurred after cessation of exercise. Time in the active work phase reflects the time to complete a search problem and provide a final trained response to alert location of the target odor. The longer a dog took to complete the search problem and alert to target odor, the shorter time until the temperature peaked in the post-work recovery phase and the dog’s temperature began to drop with passive cooling in a vehicle crate. Initially this seems counterintuitive, assuming longer duration of work would result in a longer period of continued rise in body temperature after exercise has stopped. However, it may be that the longer length of time to complete the search problem reflects dogs that worked more slowly and methodically in their search behavior, with less overall exertion in this time period. Diviero et al. observed that dogs that spent more time walking during avalanche search activity had lower mean rectal body temperatures.

### 4.4. Study Limitations

Limitations of this study include the difference in times in the three phases of work: WW: 5.83–21.00 min (15.17-min range difference); AW: 2.67–16.67 min (14-min range difference); PWR: 6.00–54.00 min (48-min range difference). These differences were allowed so as to not interfere with the dogs’ search activity, understanding that some dogs will locate and alert to the target scent more quickly than others. This reflects a common issue with field-based studies of working dogs in action, where a controlled laboratory environment is traded for a less controlled, but more realistic working environment. This range of times in the active work phase impacted peak temperature, but surprisingly in the opposite manner of what we expected, with longer active working times being associated with a shorter time to peak temperature in the post-work recovery phase. Despite the range of waiting-to-work times (15.17-min difference between shortest and longest time), there was no significant impact on body temperature based on time in this phase. In addition, a significant drop in ambient temperature (8.48 °C) occurred from the morning to afternoon iterations, making it difficult to compare body temperature responses to subsequent iterations of work.

Although the phenomenon of continual rise in body temperature following cessation of exercise is well-documented in the scientific literature [2,3,5], its importance is not readily emphasized in veterinary guidelines on prevention of canine heat injury, particularly with regard to vehicle crating following work for dogs. Gastrointestinal temperatures demonstrated by the dogs in our study were relatively low compared to previous studies, with a mean peak of only 39.66 ± 0.40 °C at the highest point in any of the three phases of work, although the highest temperature recorded of any of the dogs was 40.6 °C. These data demonstrate that a continual rise in temperature post-work occurs even when body temperatures are barely above reference ranges for clinical hyperthermia. Due to the small number of dogs and vehicles used in this study, we chose not to evaluate the impact of different types of vehicle environments on body temperature changes. It is unknown whether holding the dogs in vehicle crates immediately following exercise had an impact on the actual rate of body temperate change during post-work recovery, and whether this would have been different had the dogs returned to outdoor crates or rested on lead with their owners. However, this is common practice during training and actual search deployments and highlights that the risk for potential heat injury does not end abruptly with the cessation of active exercise or work. Further study on the impact of different types of vehicle environments on canine thermoregulation is warranted to help identify those that may facilitate or hinder cooling during post-work recovery.

Future studies should also incorporate on-site weather data collection. Although the authors attempted to gather environmental data at each site, afternoon storms compromised the location of portable weather stations making the data invalid. The authors would recommend that portable weather stations be affixed, or mounted in such a way that they cannot be easily moved, for future studies of this nature.

Field studies are frequently challenging. Future studies should incorporate controls for length of time in each phase of work, as well as measures of work intensity (such as time to alert, or activity monitors validated for such measures). As the purpose of this study was to model a typical deployment (complete with waiting and unknown search locations) the authors would encourage more investigation into factors related to deployment challenges such as stress and cumulative fatigue over multiple days of operations.

The addition of our results to the scientific base provides characterization of body temperature responses throughout the phases of waiting-to-work, active work, and post-work recovery over multiple iterations of work typical of deployment conditions. Based on these findings, along with previous studies on post-exercise rise in body temperature, we recommend that monitoring of body temperature and behaviors indicative of heat injury should continue after cessation of work until body temperature peaks and begins to decline.

## Figures and Tables

**Table 1:** Characteristic of human remains detection dogs.

Canine	Breed	Sex ^1^	Age (yr)	BW (kg)	BCS ^2^
1	Labrador Retriever	FS	5	23.58	4
2	Golden Retriever	F	1	19.50	5
3	Pit Bull Mix	MN	4	24.95	5
4	Belgian Malinois	M	2.5	30.84	5
5	Belgian Malinois	FS	8	24.49	5
6	McNab	M	1	21.77	4
7	German Shepherd	MN	8	34.93	4
8	German Shepherd	MN	6	32.20	4.5

^1^ Sex: FS = female spayed; F = female intact; MN = male neutered; M = male intact; ^2^ Nestle Purina Body Condition System, scale 1–9. BW = body weight.

**Table 2:** Environmental conditions (National Oceanic and Atmospheric Administration (NOAA), Carbondale, IL) during study period in April 2017.

Time	Ambient Temp °C	Humidity %	Wind Speed (mph)
7:52 AM	70	73	12
8:52 AM	73	71	12
9:52 AM	76	67	12
10:52 AM	77	64	12
11:52 AM	74	74	12
12:52 PM	74	76	12
1:52 PM	59	100	12
2:52 PM	60	100	0
3:52 PM	61	100	8
4:52 PM	60	100	6

**Table 3:** Mean duration, peak gastrointestinal (GI) temperature (Tgi) and change in Tgi (Tgi) of each phase of work.

Phase of Work	Duration Range (Minutes)	Mean Peak Tgi °C	Mean ∆ Tgi °C
WW	5:83–21:00	39.08	0.10
AW	2:67–16:67	39.35	0.27
PWR	6:00–54:00	39.64	0.29
Total ∆ Tgi			0.66
